# Description of the Nymphal Stages of *Hoplopleura affinis* (Anoplura: Hoplopleuridae) from the Striped Field Mouse *Apodemus agrarius* with a Global Checklist of *Hoplopleura* Species from the Genus *Apodemus*

**DOI:** 10.3390/insects13020107

**Published:** 2022-01-18

**Authors:** Paulina Kozina, Joanna N. Izdebska, Rafał Łopucki

**Affiliations:** 1 Department of Invertebrate Zoology and Parasitology, Faculty of Biology, University of Gdańsk, Wita Stwosza Street 59, 80-308 Gdansk, Poland; biojni@ug.edu.pl; 2 Centre for Interdisciplinary Research, John Paul II Catholic University of Lublin, Konstantynów 1J, 20-708 Lublin, Poland; lopucki@kul.lublin.pl

**Keywords:** sucking lice, *Hoplopleura affinis*, Anoplura, Hoplopleuridae, immature stages, first description, checklist, *Apodemus agrarius*, striped field mouse

## Abstract

**Simple Summary:**

Sucking lice are blood-feeding, external parasites of mammals. To date, approximately 540 species have been discovered, occurring in 830 hosts. New species are discovered every year, and the total number is estimated at 1500. The discovery of a species is associated with a detailed description of morphological characteristics. Most descriptions concern only adult specimens. The present study adds to the knowledge by characterizing nymphs of *Hoplopleura affinis*, a species that parasites the striped field mouse *Apodemus agrarius*, a common rodent in Europe and Africa. In addition, a checklist of *Hoplopleura* species parasitizing members of the genus *Apodemus* was compiled.

**Abstract:**

The genus *Hoplopleura* is the most speciose genus of sucking lice observed parasitizing rodents and lagomorphs (pikas). Despite the fact that the majority of Anoplura are believed to be monoxenic, many species within *Hoplopleura* may be oligoxenic. In addition, the occurrence of more than one parasite species per host species on individuals has been confirmed. As such, a precise species identification of the parasite, especially of the nymphs, is of high significance. The study is based on the material of 245 sucking louse specimens taken from 179 individuals of the striped field mouse *Apodemus agrarius* collected between 2008 and 2017. The study employs scanning microscopy to provide superior quality resolution of the studied traits. The study presents the first record of the characters of the nymphal stages of *H. affinis*, one of the common Eurasian species of the genus. Additional aspects of the biology and the host–parasite relationship of *H. affinis* are presented, e.g., female, male and nymphs of lice, showing different preferences in the choice of location (topography) on the host body. In addition, a global checklist has been made of all the species of *Hoplopleura* found parasitizing rodents of the genus *Apodemus*. Generally, the ranges of the occurrence of lice of this genus coincide with the geographic distribution of typical hosts, although this has not always been confirmed by local studies.

## 1. Introduction

Sucking lice (Psocodea: Anoplura) are hematophagous and wingless parasites of placental mammals (Mammalia: Eutheria) [1]. Most of the species have only one host throughout their life cycle, which represents the habitat for all developmental stages (eggs, three stages of nymphs, and adults). Thus far [2], 532 sucking lice species have been described, and these are known to be parasites of 830 mammal species. However, the total number of Anoplura species is estimated to be around 1500. It is believed that the majority of lice species are associated with only one host species (63%); the remaining taxa are parasites of two or more hosts; i.e., they are oligoxenic [3]. Currently, the largest genus within the Anoplura is *Hoplopleura* Enderlein, 1904 (Anoplura: Hoplopleuridae), including 176 species (136 recorded up to 1994 by Durden and Musser and 40 discovered later).

The five most common species of *Hoplopleura* in Europe are *H. acanthopus* (Burmeister, 1839), *H. affinis* (Burmeister, 1839), *H. captiosa* Johnson, 1960 (probably cosmopolitan), *H. edentula* Fahrenholz, 1916, and *H. longula* Neumann, 1909 [4]. The widest host range is shown by *H. affinis*, which is recorded from 8 species of the genus *Apodemus*, *H. acanthopus* in 5, *H. captiosa* in 2, *H. edentula* in 1, and *H. longula* was not found at all on the host of this genus [4,5,6,7,8]. 

Within *Hoplopleura*, 10 species parasitize the genus *Apodemus* Kaup, 1829 (Rodentia: Muridae). This is a Palearctic and Oriental (Eurasia, North Africa) genus, comprising 21 species. They are associated with various types of environments, from forests to open areas or arable fields, and some also enter buildings and human settlements. Some species are widespread and numerous, often recognized as crop and sanitary pests [9,10].

*Hoplopleura* species were usually identified only on the basis of adult characters (nymphs were not described for all); these characters are sometimes difficult to capture (especially when using a single identification method, e.g., only optical microscopy), which may cause identification errors, especially when studying a larger sample, where nymphal stages are also numerous. They could be assigned to the wrong species, identified on the basis of a higher probability of occurrence or alleged host specificity. This results in a misidentification of the host circle of different lice species and undermines the reliability of data on their distribution and biodiversity. Only three of the five species listed above currently have their full taxonomic characters on record, including descriptions of adult and nymphal stages (*H. acanthopus* [11], *H. captiosa* [12], *H. longula* [13]). To address this, the present study provides the first description of the nymphal stages of *H. affinis* from *Apodemus agrarius*.

## 2. Materials and Methods

### 2.1. Lice and Host Material

The material, comprising 245 specimens of *H. affinis* (50 males, 174 females, 8 first instar nymphs, 9 second instar nymphs, 4 third instar nymphs) from 179 specimens of the striped field mouse *Apodemus agrarius* Pallas, 1771 was collected between 2008 and 2017. The origin of the host specimens is provided in Table 1 (two specimens—no data available). Additionally, small mammals (common European rodents and soricomorphs) from the scientific collections of the Department of Invertebrate Zoology and Parasitology, Gdańsk, Poland, were included; *Myodes glareolus* (n = 115), *Microtus agrestis* (n = 3), *Mus musculus* (n = 292), *Apodemus flavicolis* (n = 68), *A. sylvaticus* (n = 6), and *Sorex araneus* (n = 28) were examined for *Hoplopleura* lice (no specimens of *H. affinis* found).

Where coparasitism of two sucking lice species of the genus *Hoplopleura* was observed, the individuals were not taken into account when creating nymphal stage characters due to risk of error. However, as the nymphs of *Hoplopleura* and *Polyplax* differ markedly, in cases where *H. affinis* and *Polyplax serrata* co-occurred, the nymphs were included in the identification.

The lice were collected with tweezers from ethanol-preserved rodent specimens (UGDIZP; Collection of Extant Invertebrates, University of Gdańsk, Department of Invertebrate Zoology and Parasitology, Gdańsk, Poland), by combing the coat. The lice present were kept in labeled individual vials (separately for each host) and preserved in 70% ethyl alcohol. Next, morphological structures and body surfaces of specimens were analyzed (measurements are given in mm), using two microscopic techniques:

1. Specimens intended for analysis under a light microscope were prepared by slide-mounting in polyvinyl-lactophenol [15].
2. Individuals intended for analysis with scanning electron microscopy were subjected to a series of alcohols (80–100%) and then dried in a mix of ethyl alcohol and hexamethyldisilazane (HMDS) in 1:3, 1:1, and 3:1 proportions. Finally, the specimens were transferred to pure HMDS and placed in an incubator for 24 h (37 °C) [16]. The specimens were stuck to double-sided copper tape (by Mierzejewski Materiały Samoprzylepne) and fixed on the table of a scanning electron microscope. Observations and photographs were performed with the use of Field Emission Scanning Electron Microscope JSM—7800F (manufacturer JEOL; stocked in Department of Materials Engineering and Bonding—Faculty of Mechanical Engineering, Gdańsk University of Technology, Gdańsk, Poland).

The louse specimens were deposited in the Collection of Extant Invertebrates, University of Gdańsk, Department of Invertebrate Zoology and Parasitology, Gdańsk, Poland [17].

**Figure 1:**
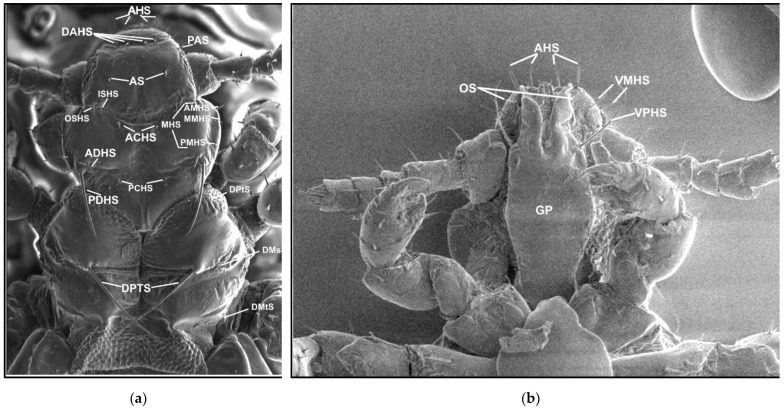
*Hoplopleura* morphological characters: (**a**) dorsal head and thorax; (**b**) ventral head. Abbreviations of cephalic and thoracic setae: ACHS, anterior central head setae; ADHS, accessory dorsal head setae; AHS, apical head setae; AMHS, anterior marginal head setae; AS, antennal setae; DAHS, dorsal anterior head setae; DMsS, dorsal mesothoracic setae; DMtS, dorsal metathoracic setae; DPTS, dorsal principal thoracic setae; DPtS, dorsal prothoracic setae; ISHS, inner sutural head setae; MHS, marginal head setae; MMHS, middle marginal head setae; OS, oral setae; OSHS, outer sutural head setae; PAS, preantennal setae; PCHS, posterior central head setae; PDHS, posterior dorsal head setae; PMHS, posterior marginal head setae; VMHS, ventral marginal head setae; VPHS, ventral principal head setae.

**Figure 2:**
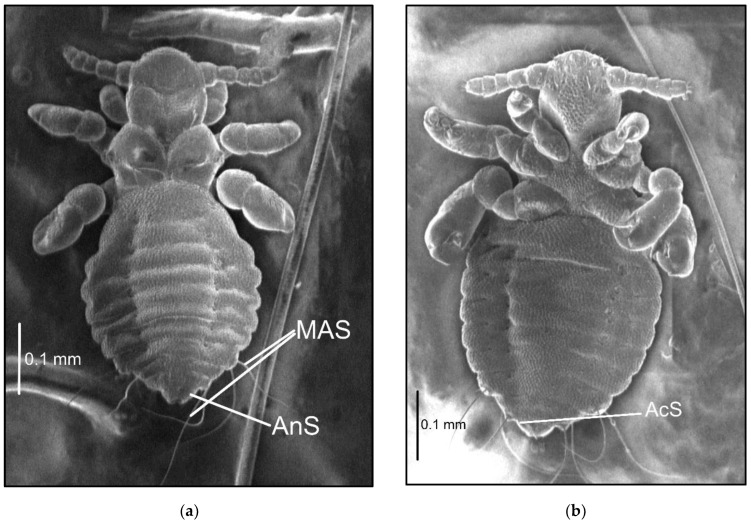
*Hoplopleura affinis* nymph III: (**a**) dorsal; (**b**) ventral. Abbreviations of abdominal setae: AcS, accessory setae; AnS, anal setae; MAS, major abdominal setae; VCAS, ventral central abdominal setae (not visible on the photo).

The names and abbreviations of individual setae or body parts are provided mostly following [1] (Figure 1 and Figure 2).

**Figure 3:**
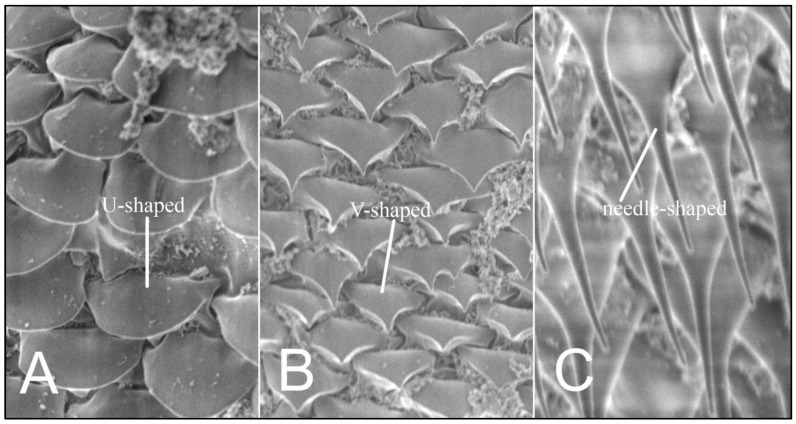
*Hoplopleura* body scales (**A**) U-shaped; (**B**) V-shaped, and (**C**) setae.

Using the scanning microscopy (SEM), three variants of ornamentation in the form of scales and setae shape are known to exist: smoothly-ended (U-shaped), sharp-ended (V-shaped), and needle-shaped setae (Figure 3).

### 2.2. The Checklist

The checklist of *Hoplopleura* species parasitizing the genus *Apodemus* has been compiled on the basis of data published during the period 1956–2019 (12 items: [4,5,6,7,10,18,19,20,21,22,23,24]. *Apodemus* species are ordered alphabetically. The scientific names, common names, and systematics of the hosts follow Wilson and Reeder [9] and the Taxonomic Information System [8].

## 3. Results

### 3.1. Description of Nymphal Stages 

**Figure 4:**
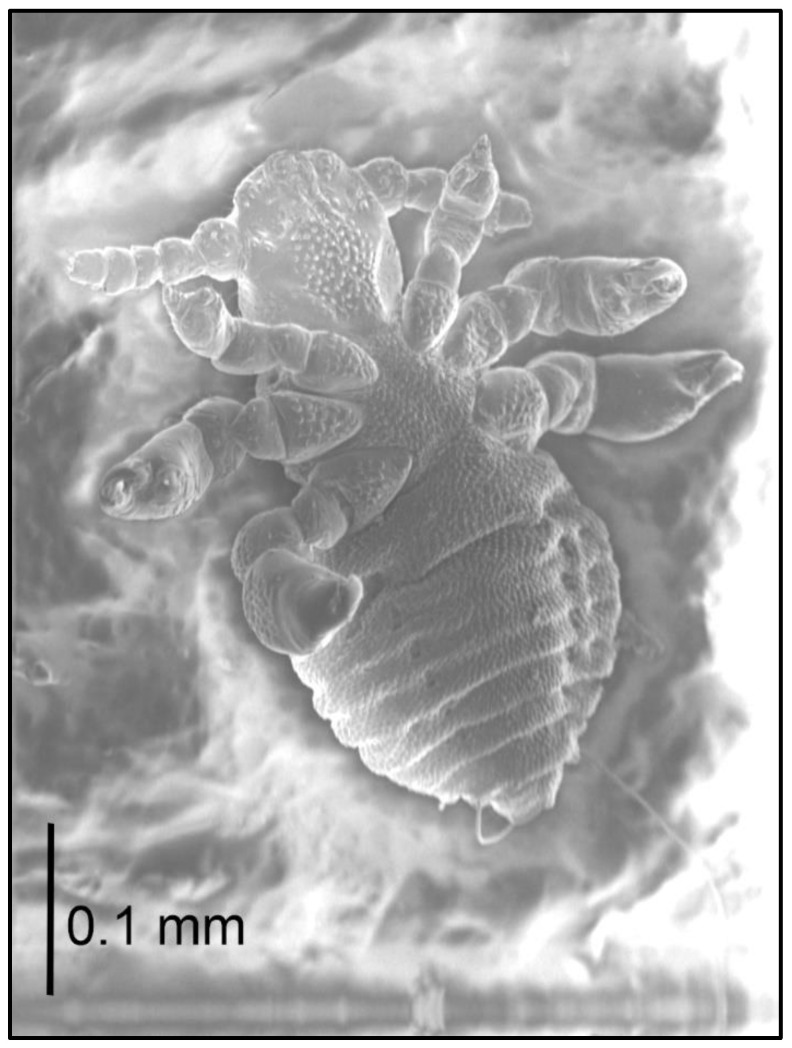
*Hoplopleura affinis* nymph I, ventral.

Nymph I (Figure 4; measurements n = 1), Body length: 0.44. Head: length 0.10, width 0.13. 

Ventral: no GP; in its place, a large number of tubercles (they form an oval field), some reminiscent of small setae in shape; three tubercles (looking like a small setae) at the base of the antennae. Head posteriorly slightly concave on both ventral and dorsal surfaces. AHS 4 in number; OS almost as long as VMHS, but thinner; above OS, there are two short bristles with a tubercle between them; VMHS measuring approx. 1/3 VPHS. Tubercles (2–4) are also on the 2nd–4th segment of antennae.

Dorsal: DAHS, PCHS, AS present; OSHS and ISHS minor, at a considerable distance from one another; MHS present, but MMHS closer to the mid-portion of the body; PDHS reaches the first thoracic segment; ADHS above the preceding one; ACHS minute.

Thorax: length 0.10, width 0.21. 

Dorsal: DPTS short, barely reaching the first thorax segment; DPtS, DMsS, and DMtS present; thorax very short and wide.

Abdomen: length 0.24, width 0.28. 

Abdomen heart-shaped, heavily wrinkled. U and V-shaped scales covering the abdomen in a tile-like manner; spiracles visible on both ventral and dorsal surfaces, heavily depressed. VCAS one pair on segment 1 (difficult to detect); MAS 2; AcS 2.

**Figure 5:**
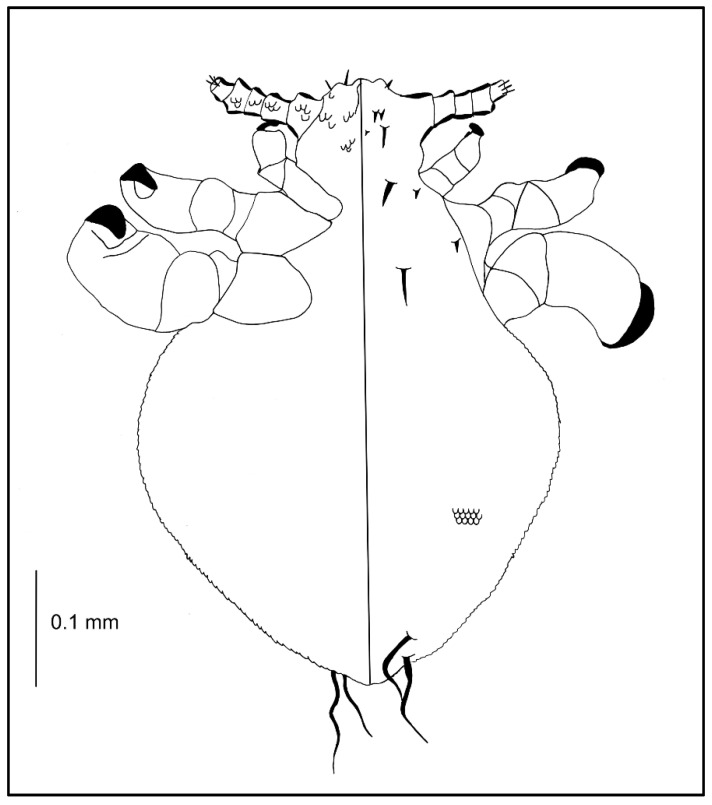
*Hoplopleura affinis* nymph II: ventral (**left**) and dorsal (**right**) view.

Nymph II (Figure 5; measurements n = 9), Body length: 0.52 (0.37–0.72). Head: length 0.10 (0.07–0.14), width 0.14 (0.12–0.15).

Ventral: convex U-shaped scales covering almost the entire surface; tubercles more flat on head corners.

Dorsal: DAHS present; OSHS and ISHS at a minor distance from one another; MHS present; PDHS reaches the first thorax segment; ADHS equal to the previous setae; ACHS minute.

Thorax: length 0.10 (0.07–0.14), width 0.14 (0.12–0.28). 

Dorsal: DPTS reaches the first abdominal segment; DPtS, DMsS, and DMtS present.

Abdomen: length 0.32 (0.21–0.14), width 0.14 (0.21–0.52).

Abdomen very broadly heart-shaped; VCAS one pair on segment 1 (difficult to detect); MAS 4 in number; AcS 2.

Nymph III (Figure 2; measurements n = 1), Body length: 0.65. Head: length 0.14, width 0.14.

Ventral: VPHS not as long; AHS, OS, and VPHS present; tubercles similar to first instar nymph; in place of GP heavily convex V-shaped tubercles present (they are arranged in the shape of a GP plate seen in adults); on the lateral corners the tubercles are flat, U-shaped. 

Dorsal: DAHS, PAS, AS, and PCHS present; OSHS and ISHS not as close to one another, very short; MMHS closer to AMHS than PMHS; PDHS barely reaches the first segment of thorax; ADHS very short and thick; whole surface covered by wide U-shaped scales (apart from the occiput); ACHS minute.

Thorax: length 0.12, width 0.22. 

Dorsal: DPTS reaches the first abdominal segment; DPtS, DMsS, and DMtS present; first part of the first segment covered thickly by scales as on the head; around the thoracic spiracle two horn-like processes, the upper larger.

Abdomen: length 0.39, width 0.34.

Abdomen heavily wrinkled, strongly covered with differently shaped scales, with well-visible spiracles (forming two lines separating the abdomen into three parts); anal area well marked, entrance heavily indented; VCAS one pair on segment 1 (difficult to detect); MAS 8 in number; AcS 2; AnS 2.

No morphological anomalies were observed in the study nymphs.

The parasite completes its entire life cycle on the host (eggs, nymphs, and adults), and feeds actively: most individuals were engorged with blood. This is confirmed by the prevalence of 36.3% and the mean intensity of 3.8 specimens (in 65 infested hosts) was recorded.

Co-occurrence of different louse species was found on one host (*Apodemus agrarius*). *Hoplopleura affinis* and *H. acanthopus* were observed in one case, and *H. affinis* and *Polyplax serrata* (Anoplura: Polyplacidae) in 16 cases.

**Figure 6:**
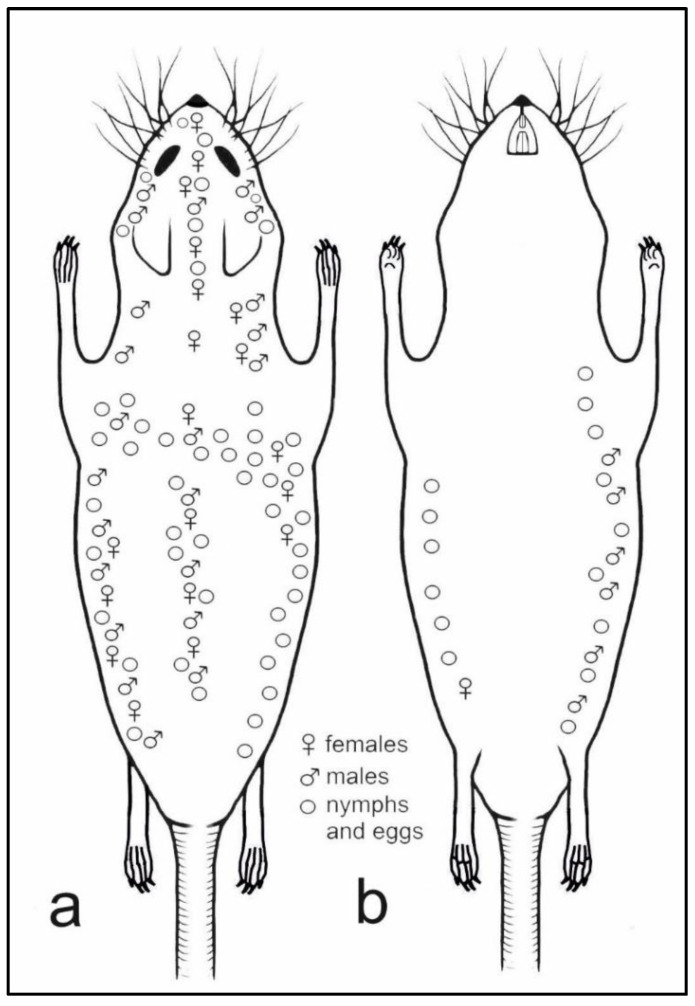
Topographic preferences of all life stages of *Hoplopleura affinis* on host body: (**a**) dorsal view; (**b**) ventral view.

Topographic preferences have been observed for *H. affinis* (Figure 6). Females (n = 119) were observed on the abdominal surface, only near the right rear leg groin. On the dorsum, however, they formed three bands, i.e., along the spine and on the lateral parts of the body, which joined on the nape of the animal. In addition, the females were observed on the head of the host animal, forming a band from the nape, between the ears up to the nose. Males (n = 27) covered the entire right side of the host animal, from the dorsum to the venter. They were found along the dorsum; they formed two bands before the forelimbs and three bands on the head, i.e., on the mid-point and on the sides of the dorsal portion, as well as between the vibrissae (upper and lower labial and jugal). Nymphs and eggs were numerous on both sides of the animal (dorsal and ventral), forming two clear bands joining on the mid-point of the dorsum. In addition, they formed three bands on the head, joining between the eyes and ears. Nymphs and eggs were rarely observed on the dorsal surface of the neck.

### 3.2. Checklist of Hoplopleura spp. on Murid Rodents of the Genus Apodemus

In 12 species of *Apodemus* mice (out of 20 known species), lice of the genus *Hoplopleura* (10 species) were recorded. Their distribution is consistent with the geographic ranges of the hosts, mainly in Eurasia and Africa (Table 2).

## 4. Discussion

The morphology of *H. affinis* nymphs considerably differs from other European species (*H. acanthopus*—Holarctic parasite on voles, *H. captiosa*—cosmopolitan on *M. musculus*, *H. edentula*—Eurasia, *H. longula*—Eurasia) (Table 2). No morphological characters of *H. edentula* nymphs exists. The characteristic, heart-shaped body, is a feature that distinguishes the nymphs of *H. affinis* from the other three mentioned above. *H. affinis* has a heart-shaped, more or less broad, corrugated abdomen, while *H. acanthopus*, *H. captiosa,* and *H. longula* have an elongated, egg-shaped or barrel-shaped abdomen with poorly or without corrugation [11,12,13]. 

Nymphs of *H. longula* have very long posterior dorsal head setae (PDHS), extending to the second abdominal segment. PDHS of *H. affinis* never reach the second abdominal segment [13]. 

The tubercles cover the upper part of the head of *H. acanthopus*, on *H. affinis* they additionally form a field along the middle part of the head (in place of the gular plate GP), while on the head of *H. longula* they form a cross-shape. The tubercles on ventral surface of head and antennae *H. captiosa* are blunt, *H. affinis*- line small setae in shape (V-shaped) [11,12,13]. 

Nymphs of *H. affinis* can be easily distinguished from one another: nymph I has two major abdominal setae (MAS) and two accessory setae (AcS); nymph II has four major abdominal setae and two accessory setae; nymph III—eight major abdominal setae, two accessory setae and two anal setae (AnS). 

The nymphal stages of *H. affinis* demonstrate a similar heart-shaped body to *H. malabarica* and *H. sicata*, but these parasites were not found on *Apodemus* [26].

Anomalies in morphology (differences in the number and size of the setae on the sternal plates of the abdomen and irregularities in the structure of the plate itself) were observed for the adult examined individuals [27].

*Hoplopleura affinis* appears to be a typical parasite of *A*. *agrarius* in Europe; despite extensive research, it has not been found on *Myodes glareolus*, *Microtus agrestis*, *Mus musculus*, *Apodemus sylvaticus,* or *Sorex araneus* [4,25,28,29,30]. Other results indicate this as well: occurrence of all life stages on the host, observation of blood-filled individuals (this study).

Few studies provide the locations of the sucking lice on the host body. However, Dubinin [31] notes that for *H. affinis* parasitizing *A*. *agrarius*, the lice were located only on the dorsal head and a small neighboring part of the neck. The current study provides more extensive knowledge, with the louse being found to cover a larger preferred area, reaching the posterior limbs of the host. There are also no data on the occurrence of *H. affinis* nymphs and eggs present on *A*. *agrarius* in the available literature. The present study adds to the knowledge, with multiple occurrences on the dorsal and ventral parts of the animal. No differences were observed in topographic preferences for different life stages of *H. affinis*. One common feature is evident: none of the stages occurred on the ventral head. Perhaps this is due to the structure of the host’s coat, the hairs are sparser and shorter there [32] and specific behavior patterns, e.g., self-grooming or self-cleaning behaviors, when rodents self-groom by scratching to clean or groom the fur [33,34].

Analyzing the global checklist of *Hoplopleura* species of the genus *Apodemus* (Table 2), it can be seen that the absence of sucking lice findings in a given host is mainly due to its status: endemic, difficult to access or recently described species. This applies, for example, to *A. alpicola*, a poorly understood species discovered in the mid-twentieth century, endemic to the alpine region, and often inhabiting hard-to-reach protected areas. Similarly, poorly known is *A. hyrcanicus*, described only in 1992, limited in range to forest regions from the southern Caucasus to Central Asia, moreover, usually not sympatric with either *Apodemus* [9], which also limits the transmission of parasites. In contrast, in widely distributed and much better-studied species, such as *A. agrarius*, *A. flavicollis*, *A. sylvaticus*, and *A. uralensis*, there are usually several, also usually widespread, *Hoplopleura* species.

## 5. Conclusions

Research on sucking lice (Anoplura) has so far been carried out selectively, focusing mainly on their pathogenicity and related aspects. Descriptions of new species are usually based only on adults. The lack of knowledge of the juvenile stages generate false data on the host specificity of individual parasite species, their host circle, transfer possibilities between hosts, geographical distribution, or habitat preferences. Consequently, it leads to false conclusions regarding various aspects of the functioning of the parasite–host systems, the spread and transmission possibilities of parasites, and in the case of particularly pathogenic parasites or pathogens vectors, their health significance. The issue of the correct identification of the stages of juvenile parasites is currently one of the universal problems of parasitology. This especially applies to the phases of the life cycles, where juvenile stages are the dominant group in the structure of the parasite population, or mature stages, which are usually the basis for species identification, do not occur at all. An example is the parasitic nematodes of the Anisakidae family with complex life cycles where only larval stages are present in intermediate/paratenic hosts (fish). They are of zoonotic importance as they can be invasive and pathogenic to humans [35,36,37]. Problems with identifying the larval stages may also concern various ticks important as vectors of pathogens [38]. The lack of descriptions for nymphs causes them to be mistakenly assigned to species and consequently to host species. It also causes a lack of recognition the structure and dynamics of the population development of the lice species within the host, as well as the seasonal dynamics. Hence, it is important to describe the nymphal stages as well. The current study adds to the knowledge on this subject by providing descriptions for the nymphs of *H. affinis*. It should be kept in mind that assigning nymphs to a given species is extremely difficult and requires the consideration of a number of inter-related criteria, including the presence of adult stages and exclusion of coparasitism with similar species.

## Figures and Tables

**Table 1:** Host and lice specimen data from Poland (physico-geographical regions follow [14]).

Physico-Geographical Region	*Apodemus agrarius* Collection Numbers	Location			*Hoplopleura affinis* Collection Numbers	Comments
		City	Voivodeship	GPS Data		
CENTRAL EUROPEAN LOWLAND						
Central Poland Lowlands	2 specimens (UGDIZPMRMAa122K, UGDIZPMRMAa123K)	Niemce	Lublin	51°21′ N 22°37′ E	11 males (UGDIZPMAaAHHaf1m-UGDIZPMAgHaf11m)	2 cases of co-occurrence of *H. affinis*-*P. serrata*
					36 females (UGDIZPMAaAHHaf12f-UGDIZPMAaAHHaf47f)	
					1 first instar nymph (UGDIZPMAaAHHaf48N1),	
					1 third instar nymph (UGDIZPMAaAHHaf49N3)	
Southern Baltic Coastlands	6 specimens (UGDIZPMRMAa156K, UGDIZPMRMAa157K, UGDIZPMRMAa161K-UGDIZPMRMAa163K, UGDIZPMRMAa177K)	Arciszewo	Pomeranian	54°15′ N 18°33′ E	1 female (UGDIZPMAaAHHaf50f)	
	4 specimens (UGDIZPMRMAa4K, UGDIZPMRMAa172K-UGDIZPMRMAa174K)	Gdynia	Pomeranian	54°29′ N 18°25′ E	2 males (UGDIZPMAaAHHaf51m, UGDIZPMAaAHHaf52m)	Co-occurrence of *H. affinis*-*H. acanthopus*
					15 females (UGDIZPMAaAHHaf53f- UGDIZPMAaAHHaf67f)	
					1 third instar nymph (UGDIZPMAaAHHaf68N3)	measurements—light microscopy; photos—scanning microscopy
	1 specimen (UGDIZPMRMAa151K)	Gdańsk 1	Pomeranian	54°22′ N 18°36′ E	3 females (UGDIZPMAaAHHaf69f-UGDIZPMAaAHHaf71f)	
	1 specimen (UGDIZPMRMAa155K)	Gdańsk 2	Pomeranian	54°25′ N 18°36′ E	no specimens	
	1 specimen (UGDIZPMRMAa178K)	Hopowo	Pomeranian	54°15′ N 18°14′ E	1 female (UGDIZPMAaAHHaf72f)	
	1 specimen (UGDIZPMRMAa179K)	Łebieniec	Pomeranian	54°43′ N 17°36′ E	7 males (UGDIZPMAaAHHaf73m- UGDIZPMAaAHHaf79m)	
					21 females (UGDIZPMAaAHHaf80f- UGDIZPMAaAHHaf100f)	
					1 first instar nymph (UGDIZPMAaAHHaf101N1)	measurements
					7 s instar nymphs (UGDIZPMAaAHHaf102N2- UGDIZPMAaAHHaf108N2)	measurements
	2 specimens (UGDIZPMRMAa175K, UGDIZPMRMAa176K)	Swarożyn	Pomeranian	54°02′ N 18°39′ E	no specimens	
	48 specimens (UGDIZPMRMAa5K- UGDIZPMRMAa50K; UGDIZPMRMAa170K, UGDIZPMRMAa171K)	Tczew	Pomeranian	54°06′ N 18°46′ E	1 male (UGDIZPMAaAHHaf109m)	
CZECH MASSIF						
Sudety Mts and Sudety Foreland	3 specimens (UGDIZPMRMAa1K- UGDIZPMRMAa3K)	Nowa Ruda	Lower Silesia	50°31′ N 16°33′ E	no specimens	
POLISH UPLANDS						
Lublin-Lviv Upland	11 specimens (UGDIZPMRMAa66K- UGDIZPMRMAa73K, UGDIZPMRMAa76K, UGDIZPMRMAa127K, UGDIZPMRMAa128K)	Kijany	Lublin	51°19′ N 22°46′ E	2 males (UGDIZPMAaAHHaf110m, UGDIZPMAaAHHaf111m)	2 cases of co-occurrence of *H. affinis*-*P. serrata*
					22 females (UGDIZPMAaAHHaf112f- UGDIZPMAaAHHaf133f)	
					1 first instar nymph (UGDIZPMAaAHHaf134N1)	
	26 specimens (UGDIZPMRMAa86K, UGDIZPMRMAa87K, UGDIZPMRMAa89K- UGDIZPMRMAa91K, UGDIZPMRMAa93K- UGDIZPMRMAa114K)	Łęczna	Lublin	51°18′ N 22°52′ E	5 males (UGDIZPMAaAHHaf135m- UGDIZPMAaAHHaf139m)	2 cases of co-occurrence of *H. affinis*-*P. serrata*
					23 females (UGDIZPMAaAHHaf140f- UGDIZPMAaAHHaf162f)	
					4 first instar nymphs (UGDIZPMAaAHHaf163N1- UGDIZPMAaAHHaf166N1)	
					1 s instar nymph (UGDIZPMAaAHHaf167N2)	measurements—light microscopy
					2 third instar nymphs (UGDIZPMAaAHHaf168N3, UGDIZPMAaAHHaf169N3)	
	10 specimens (UGDIZPMRMAa51K- UGDIZPMRMAa60K)	Nowogród	Lublin	51°19′ N 22°47′ E	3 males (UGDIZPMAaAHHaf170m-UGDIZPMAaAHHaf172m)	Co-occurrence of *H. affinis*-*P. serrata*
					3 females (UGDIZPMAaAHHaf173f- UGDIZPMAaAHHaf175f)	
WESTERN CARPATHIANS and WESTERN AND NORTHERN SUBCARPATHIANS						
Outer Western Carpathians	19 specimens (UGDIZPMRMAa61K- UGDIZPMRMAa65K, UGDIZPMRMAa74K, UGDIZPMRMAa75K, UGDIZPMRMAa77K- UGDIZPMRMAa85K, UGDIZPMRMAa88K, UGDIZPMRMAa92K, UGDIZPMRMAa121K)	Rymanów	Subcarpathian	49°35′ N 21°50′ E	10 males (UGDIZPMAaAHHaf176m- UGDIZPMAaAHHaf185m)	2 cases of co-occurrence of *H. affinis*-*P. serrata*
					31 females (UGDIZPMAaAHHaf186f- UGDIZPMAaAHHaf216f)	
					1 first instar nymph (UGDIZPMAaAHHaf217N1)	
					1 s instar nymph (UGDIZPMAaAHHaf218N2)	measurements—light microscopy; photos—scanning microscopy
EASTERN BALTIC-BELARUS LOWLAND						
	14 specimens (UGDIZPMRMAa115K- UGDIZPMRMAa120K, UGDIZPMRMAa124K, UGDIZPMRMAa125K, UGDIZPMRMAa136K, UGDIZPMRMAa137K, UGDIZPMRMAa140K, UGDIZPMRMAa141K, UGDIZPMRMAa149K, UGDIZPMRMAa150K)	Chełm	Lublin	51°07′ N 23°28′ E	3 males (UGDIZPMAaAHHaf219m- UGDIZPMAaAHHaf221m)	3 cases of co-occurrence of *H. affinis*-*P. serrata*
					6 females (UGDIZPMAaAHHaf222f- UGDIZPMAaAHHaf227f)	
	17 specimens (UGDIZPMRMAa126K, UGDIZPMRMAa129K- UGDIZPMRMAa135K, UGDIZPMRMAa138K, UGDIZPMRMAa139K, UGDIZPMRMAa142K- UGDIZPMRMAa148K)	Strupin Duży	Lublin	51°05′ N 23°30′ E	5 males (UGDIZPMAaAHHaf228m-UGDIZPMAaAHHaf232m)	4 cases of co-occurrence of *H. affinis*-*P. serrata*
					12 females (UGDIZPMAaAHHaf233f- UGDIZPMAaAHHaf244f)	
Eastern Baltic Coastland	3 specimens (UGDIZPMRMAa164K, UGDIZPMRMAa168K, UGDIZPMRMAa169K)	Stare Jabłonki	Warmian-Masurian	53°42′ N 20°04′ E	no specimens	
	4 specimens (UGDIZPMRMAa152K, UGDIZPMRMAa153K, UGDIZPMRMAa165K, UGDIZPMRMAa166K)	Kleszczewo	Greater Poland	54°11′ N 18°30′ E	no specimens	
	4 specimens (UGDIZPMRMAa154K, UGDIZPMRMAa158K-UGDIZPMRMAa160K)	Słomowo	Greater Poland	52°21′ N 17°32′ E	1 male (UGDIZPMAaAHHaf245m)	
no data					UGDIZPMAaAHHaf246f	
					UGDIZPMAaAHHaf247f	

**Table 2:** List of *Hoplopleura* species parasitizing members of the genus *Apodemus*. Geographic distributions of both *Apodemus* and *Hoplopleura* species are provided.

Host	Host Distribution	*Hoplopleura* Species	Parasite Distribution
*Apodemus agrarius* (Pallas, 1771)	Eastern Europe to Eastern Asia	*H. acanthopus* [6,7,25]	Holarctic region ^1^
		*H. affinis* [2,4,6,7,25]	Eurasia
*Apodemus alpicola* Heinrich, 1952	endemic to North West parts of the Alps	no data	no data
*Apodemus argenteus* Temminck, 1844	endemic to Japan	*H. akanezumi* [6]	Japan, Taiwan
		*H. himenezumi* [2,4,6,20]	Japan
		*H. inagakii* [6]	Japan
*Apodemus chevrieri* Milne-Edwards, 1868	West Central China	*H. affinis* [2,4]	Eurasia
*Apodemus draco* Barrett-Hamilton, 1900 = *Apodemus ilex* Thomas, 1922	China and North East India	no data	no data
*Apodemus epimelas* (Nehring, 1902)	South Eastern Europe	no data	no data
*Apodemus flavicolis* (Melchior, 1834)	Europe and western Asia	*H. acanthopus* [6,7,22,23,25]	Holarctic region ^1^
		*H. affinis* [2,4,7]	Eurasia
		*H. edentula* [7]	Eurasia
		*H. himalayana* [4,6,21]	India
*Apodemus gurkha* Thomas, 1924	endemic to Nepal	*H. pacifica* [5]	cosmopolitan (in tropical, subtropical and southern temperature zones) ^2^
*Apodemus hyrcanicus* Vorontsov, Boyeskorov, and Mezhzherin, 1992	South Caucasus to Central Asia	no data	no data
*Apodemus latronum* Thomas, 1911	China, India and Burma	no data	no data
*Apodemus mystacinus* Danford and Alston, 1877	Albania, Bosnia and Herzegovina, Croatia, Georgia, Greece, Iran, Iraq, Israel, Jordan, Lebanon, Saudi Arabia, Serbia and Montenegro	no data	no data
*Apodemus pallipes* Barrett-Hamilton, 1900 = *Apodemus wardi* (Wroughton, 1908)	Kyrgyzstan, Tajikistan, Afghanistan, India, Iran, Nepal and Pakistan	*H. affinis* [5]	Eurasia
		*H. captiosa* [5]	cosmopolitan (probably) ^3^
		*H. himalayana* [2,4]	India
		*H. pacifica* [5]	cosmopolitan (in tropical, subtropical and southern temperature zones) ^2^
*Apodemus peninsulae* Thomas, 1906	Northeastern Asia, including the Russian Far East, northern China, the Korean Peninsula, Sakhalin and Hokkaidō	no data	no data
*Apodemus ponticus* Sviridenko, 1936	endemic to the Caucasus	no data	no data
*Apodemus rusiges* Miller, 1913	India, Nepal and Pakistan	*H. himalayana* [2,4]	India
*Apodemus semotus* Thomas, 1908	endemic to Taiwan	*H. akanezumi* [2,4]	Japan ^4^, Taiwan
*Apodemus speciosus* Temminck, 1844	endemic to Japan	*H. acanthopus* [6]	Holarctic region ^1^
		*H. affinis* [2,4]	Eurasia
		*H. akanezumi* [2,4,6]	Japan, Taiwan
		*H. himenezumi* [6]	Japan
		*H. inagakii* [6]	Japan
*Apodemus sylvaticus* Linnaeus, 1758	Europe and North Western Africa	*H. acanthopus* [6,22,23,25]	Holarctic region ^1^
		*H. affinis* [2,4,6,24,25]	Eurasia
		*H. captiosa* [25]	cosmopolitan (probably) ^3^
*Apodemus uralensis* (Pallas, 1811) = *A. microps* Kratochvíl and Rosicky, 1952	Central Europe and Asia	*H. acanthopus* [6,7]	Holarctic region ^1^
		*H. affinis* [7]	Eurasia
*Apodemus witherbyi* Thomas, 1902	Eastern Europe, Near East and Central Asia	*H. affinis* [8]	Eurasia

^1^ typical hosts—voles. ^2^ typical hosts—*Rattus* spp. ^3^ cosmopolitan on *Mus musculus*, typical host. ^4^ on *A. speciosus.*

## Data Availability

The data presented are available in this article.
